# Ensemble learning-based feature selection for phosphorylation site detection

**DOI:** 10.3389/fgene.2022.984068

**Published:** 2022-10-21

**Authors:** Songbo Liu, Chengmin Cui, Huipeng Chen, Tong Liu

**Affiliations:** ^1^ School of Computer Science and Technology, Harbin Institute of Technology, Harbin, China; ^2^ Beijing Institute of Control Engineering, China Academy of Space Technology, Beijing, China

**Keywords:** phosphorylation site, ensemble learning (EN), marchine-learning, feature selection (FS), SARS-cov-2

## Abstract

SARS-COV-2 is prevalent all over the world, causing more than six million deaths and seriously affecting human health. At present, there is no specific drug against SARS-COV-2. Protein phosphorylation is an important way to understand the mechanism of SARS -COV-2 infection. It is often expensive and time-consuming to identify phosphorylation sites with specific modified residues through experiments. A method that uses machine learning to make predictions about them is proposed. As all the methods of extracting protein sequence features are knowledge-driven, these features may not be effective for detecting phosphorylation sites without a complete understanding of the mechanism of protein. Moreover, redundant features also have a great impact on the fitting degree of the model. To solve these problems, we propose a feature selection method based on ensemble learning, which firstly extracts protein sequence features based on knowledge, then quantifies the importance score of each feature based on data, and finally uses the subset of important features as the final features to predict phosphorylation sites.

## 1 Introduction

According to the World Health Organization (WHO), Severe Acute Respiratory Syndrome Coronavirus 2 (SARS-COV-2), a novel coronavirus known as Coronavirus Disease 2019 (Covid-19) infection causing coronavirus disease, is the key viruses of the pandemic. As of June 2022, there are already a total of 531 million confirmed cases and up to 6.3 million deaths worldwide. The disease is causing tremendous stress and tension not only in global healthcare systems, but in a variety of fields. And the impact of the virus far exceeds that of SARS in 2003 (Cui et al., 2003; Read et al., 2021). Although vaccination against SARS-COV-2 is now available, the virus cannot be completely eradicated nowadays due to the huge global population base and the rapid mutation of the virus, and infection with the novel coronavirus remains severe in most regions (Cai et al., 2021a; Cai et al., 2021b; Li T. et al., 2021; Song et al., 2021). In response to the COVID-19 epidemic, the search for potential viral genetic or protein information as soon as possible will greatly help clinicians to improve diagnostic and therapeutic efficiency and contribute to the development of more effective treatments. The level of investment in vaccine and drug development is high, so a comprehensive understanding of the molecular mechanisms of SARS-COV-2 infection and changes in host cellular pathways is essential for rational drug design (Li F. et al., 2021; Ren et al., 2021; Tang et al., 2021).

Phosphorylation is one of the most important cellular biological processes, who is involved in signaling of various processes, including cell cycle, proliferation and apoptosis (Hunter, 1998; Lawlor and Alessi, 2001; Cohen, 2002). During phosphorylation, a phosphate group is added to the side chain of an amino acid, mainly serine (Ser, S), threonine (Thr, T) or tyrosine (Tyr, Y), but to a lesser extent to arginine, lysine and histidine residues (Pearson and Kemp, 1991). Studies have shown that phosphorylation occurs in 30–50% of all proteins (Pinna and Ruzzene, 1996). Therefore, accurate prediction of phosphorylation sites of proteins may help to understand the overall intracellular activity.

With the development of high-throughput sequencing, the functions of many phosphorylation sites are well annotated. Regulated kinases can be easily identified from phosphorylation sequence, many of which may have become drug targets with therapeutic potential (Ochoa et al., 2016; Ochoa et al., 2020). Bouhaddou et al. presented a quantitative mass spectrometry-based phosphorylated protein proteomics study that investigated SARS-COV-2 infection in cells, revealing a reorganization of host and viral protein phosphorylation (Bouhaddou et al., 2020). Hekman et al. performed a quantitative phosphorylated protein proteomics study of SARS-COV-2 infection to find the connection (Hekman et al., 2020). Due to the biological importance of protein kinases in cell signaling and the steadily increasing number of reports identifying phosphorylation sites (Knight et al., 2003), it has become impractical for experimental molecular biologists to track all phosphorylation modifications of proteins in their field of study. Most of the experimental require expensive equipment and labor.

Therefore, machine learning methods based on high-throughput obtained sequencing data are heavily used. QUOKKA applied multiple sequence scoring functions in combination with optimized logistic regression algorithms to predict phosphorylation sites (Li et al., 2018). PhosPred-RF (Wei et al., 2017) and PhosphoSVM (Dou et al., 2014), used only sequence-based features for random forest (RF) and support vector machine (SVM) based predictions, respectively. PhosphoPredict (Song et al., 2017) also used a combination of sequence and functional features to decipher kinase-specific substrates and their associated phosphorylation sites. Lv et al. used word vectors to extract features and LSTM network architecture for phosphorylation site identification (Lv et al., 2021).

**FIGURE 1:**
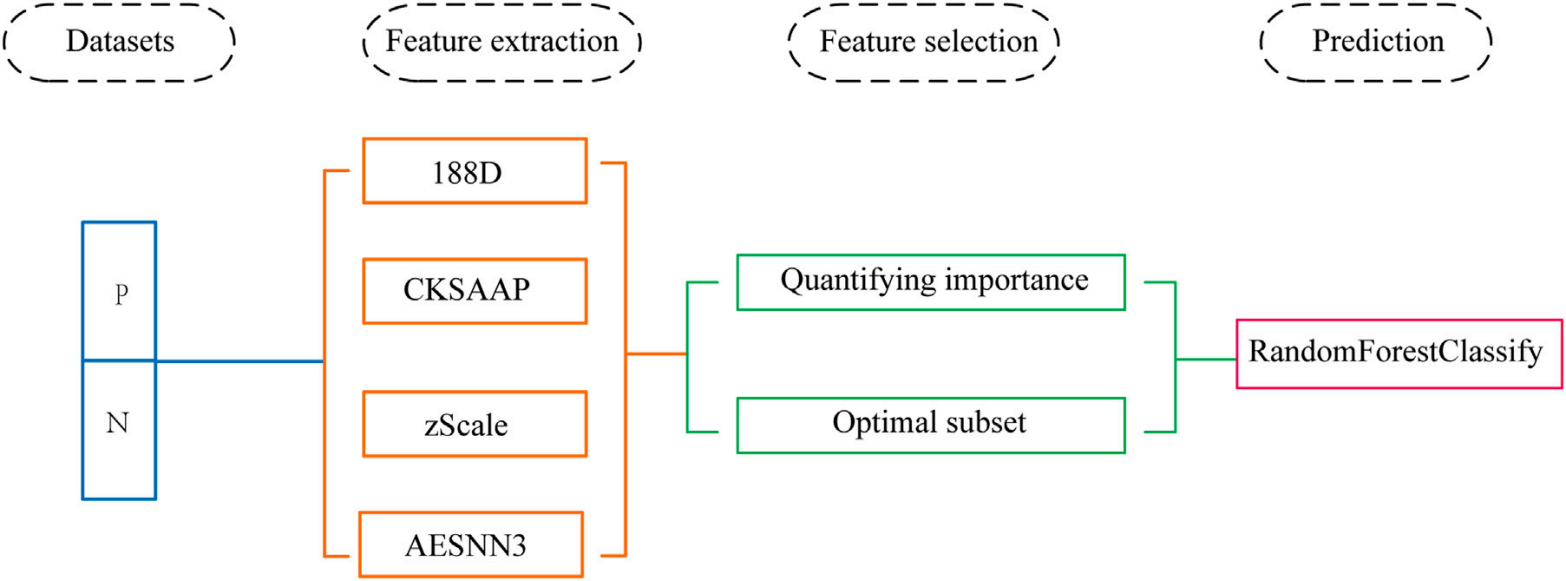
The method flowchart.

In this paper, we use machine learning techniques to predict the phosphorylation sites of SARS-CoV-2 based on protein sequences combined with amino acid composition, physicochemical properties and zScale and AESNN3 features. The problem has been conversion to a two-class classification problem, where the two classes correspond to phosphorylation sites and non-phosphorylation sites, respectively. We quantify the importance of each feature component to select a subset of features as a preprocessing step. After feature dimensional reduction, we use the Random Forest algorithm based on ensemble learning to make predictions for phosphorylation sites. We outperform other algorithms in accuracy and number of features in independent test datasets. Figure 1 shows the complete data processing approach.

## 2 Methods

### 2.1 Data

In this study, we use experimentally validated phosphorylation site data from human A549 cells infected with SARS-COV-2 (Stukalov, 2021), which consisted of 14,119 phosphorylation sites. Since sequences are often affected by homology and redundancy issues. Therefore, eliminating sequence redundancy and reducing homologous sequences are prerequisites for understanding the dataset and preventing overfitting of the model. To deal with this effect, Hao et al. used CD-HIT (Li and Godzik, 2006) to remove homologous sequences with 30% parameters as the threshold, and truncated the sequences into 33 residues long sequences centered on S/T and Y sites, so as to compare them with other phosphorylation prediction methods. To balance the amount of positive and negative sample data, Hao et al. randomly selected a subset of non-redundant negative samples to match the number of positive samples (Basith et al., 2020; Mei et al., 2021; Wei et al., 2021). A fragment is defined as a positive sample if its centers S/T and Y are phosphorylation, otherwise the sample is considered negative. 20% is used as independent test data. The final S/T and Y data are obtained in Table 1.

**TABLE 1:** Phosphorylation data collected in this study.

Data type	Residue type	Positive samples	Negative samples
Train	S/T	4308	4308
	Y	81	81
Test	S/T	1079	1079
	Y	21	21

### 2.2 Feature extraction

In general, machine learning cannot directly process sequence data, so protein sequences need to be encoded. The commonly used feature extraction methods, depending on the diversity of protein, are Amino Acid Composition (AAC) based on the type and content of amino acids, the physicochemical properties of amino acid, and n-skip-gram based on the simple arrangement order of amino acids. In this study, we mainly use these feature extraction methods of amino acid content, physicochemical properties and arrangement order. To convenience the description, the protein sequence is defined as:

$$\begin{array}{c} P=p_{1}p_{2}p_{3}\ldots p_{L}\;,\;p_{i}\in \left\{A,\;C,\;D,\;\ldots Y\right\}, \end{array}$$

where P is the sequence of a sample, $p_{i}$ is the $i_{th}$ amino acid in the sequence, and L is the length of sequence.

#### 2.2.1 AAC

Amino Acid Composition (AAC) is a relatively simple protein feature extraction method, which codes the percentage of each amino acid type in protein sequence. The AAC feature of the sequence of an amino acid sample is as follows:

$$\begin{array}{c} AAC=\frac{N\left(p_{i}\right)}{L}\;,\;0<i\leq 20, \end{array}$$

where $N\left(p_{i}\right)$ represents the number of $p_{i}$ in the sequence, and L represents the length of the sequence. Finally, the dimension of this feature is 20 × 1.

#### 2.2.2 CTD

Composition (C), Transition (T) and Distribution (D) represent the amino acid physicochemical features of each sequence (Govindan and Nair, 2013). This feature divides amino acids into three grades according to certain properties, with positive, neutral, negative. C is the percentage of each grade. T features describe three transitional relationships between residue pairs, i.e., a negative residue followed by a neutral; a positive residue followed by a negative; a positive residue followed by a neutral. D refers to the ratio of the first, 25%, 50%, 75% and the last of amino acid residues of three levels in each group of amino acids and the length of the whole protein sequence. CTD is a method without sequence alignment, and its effectiveness depends largely on the amino acid classification.

#### 2.2.3 188D

Cai et al. (2003) proposed a physicochemical property-based 188D feature based on an extended combination of AAC and CTD. In this feature, the first 20 features are amino acid composition, and the percentage of 20 amino acids extracted from AAC feature types. The remaining 168 features describe eight physicochemical properties of amino acids, including hydrophobicity, normalized van der Waals volume, polarity, polarizability, charge, surface tension, secondary structure, and solvent accessibility (Govindan and Nair, 2013; Lin et al., 2013; Zou et al., 2013). For each property, according to the CTD principle, it is divided into three levels, and then the Composition, Transition and Distribution of the property are calculated, and each physicochemical property gets a 21-dimensional numerical feature description. Eight physicochemical properties are calculated separately and the results are spliced, and finally 168 features are obtained.

#### 2.2.4 CKSAAP

Composition of K-Spaced Acid Pairs (CKSAAP) encodes the proportion of amino acid pairs separated by k residues and is used to characterize the amino acid composition background of the sequence surrounding the post-translational modification site. This feature takes into account both sequence and quantity information of amino acids and is defined as follows:

$$\begin{array}{c} CKSAAP=\frac{N_{p_{i}p_{j}}}{L},\;p_{i},\;p_{j}\in \left\{A,C,D,\ldots ,Y\right\} \end{array}$$

j=i+k+1, i,j≤L

where $N_{p_{i}p_{j}}$ is the content of $p_{i}p_{j}$ residue pairs and L is the length of the amino acid sequence. In this study we use k=1.

#### 2.2.5 zScale

zScale converts amino acid sequences into five physicochemical descriptor variables for feature, which are developed by Sandberg et al., in 1998 (Sandberg et al., 1998), which describe the lipophilic, steric and electronic descriptors of amino acids, and then is dimensionality reduced using the PCA method. The zScale descriptor is used to encode peptides of equal length.

#### 2.2.6 AESNN3

AESNN3 is developed by Lin et al. who apply an artificial neural network approach to compare protein structures (Lin et al., 2002; Liu et al., 2019). They encode each amino acid sequence in 3-dimensional space and find that AESNN3 vector expression is the best expression method for studying proteins using neural network methods.

### 2.3 Feature selection

Protein function is a combination of the type, number, and sequence of amino acids and the spatial structure of the peptide chain. Although a great deal of research has been done on the function of proteins today, there are still many gaps in the relationship between function and sequence. Moreover, in our selected feature extraction methods, there are not necessarily features related to protein phosphorylation. Therefore, the extracted features need to be filtered, and fewer features are also more effective in characterizing protein phosphorylation in a more fundamental way. Here we propose a feature selection method based on ensembled learning as follows.

In order to find out the important features, we score each feature component according to its degree of influence on the classification result. The steps are as follows. Firstly, we assume that all the extracted features are valid for model classification. Then we randomly select 70% of the training data for training the classifier and calculate the classification accuracy by using the model as $score_{1}$ in the out-of-bag data. Then we iterate through each feature component and if the feature component is important for identifying phosphorylated sites, then adding interference to the feature component will have a great impact on the classification results. Based on this idea, we randomly disrupted the out-of-bag data with one-dimensional feature component to ensure consistent data distribution, and used the classifier trained at 70% to predict the out-of-bag data after disrupting the one-dimensional feature component to obtain the classification accuracy $score_{2}$ . The importance score of the feature is defined as

$$\begin{array}{c} vimps_{i}=score_{1}-score_{2}. \end{array}$$

To find the optimal subset of features for each type, we train the classifier by order accumulating features on the training data set based on the importance scores of the features, and calculate the classification accuracy on the test data. Since some features have no useful information for the classification, the classification accuracy is incremented and then smoothed when the features are added according to the incremental importance score method. And we select the features with higher accuracy and fewer dimensions of features as the optimal feature subset. We perform feature selection for each type of feature in turn and stitch the optimal feature subset.

### 2.4 Ensemble classifier

Traditional classifiers are sensitive to the distribution of data, but it is difficult to calculate the distribution of the high-dimensional data. Therefore, we use multiple classifiers to train the data at the same time, and then find the best one to calculate the feature importance. Here we use common classifiers, k-neighbors classifier (KNN), support vector machine (SVM), logistic regression (LR), multilayer perceptron (MLP), Gaussian naive bayes (GNB), decision tree classifier (DTC), for ensemble (Pedregosa et al., 2011). In order to make the method more applicable, we use default parameters for fitting the data. We divide the training data, part of which is used to train all classifiers, and part of which is to choose the classifier that fits the data best. Then the optimal classifier is used to predict the phosphorylation.

In this paper, various classifiers are used and the corresponding classification accuracies are calculated, and the classifier with best accuracy is recorded. This procedure is repeated for 100 times. The decision tree classifier is the most selected classifier. Therefore, we use the random forest method as the final classifier for predict the phosphorylation.

Because of the ensembled classifier used, its time complexity will increase, but the time consumption is worth it.

Measurements.

In this paper, in addition to using the common measure ACC to assess the effectiveness of classification models, we also use sensitivity (SN), specificity (SP), which is defined as follows

$$ACC=\frac{TP+TN}{TP+TN+FP+FN},$$

$$\begin{array}{c} SN=\frac{TP}{TP+FN}, \end{array}$$

$$SP=\frac{TN}{TN+FP}\;,$$

where TP, FP, TN and FN respectively represent true positive, false positive, true negative and false negative.

## 3 Result

We first extract four types of features based on the sequence of the protein, and then find the optimal subset of each feature separately. After finding the feature subsets, we use the test set to calculate the classification accuracy on the optimal subsets, and then briefly analyze the important features. Finally, we splice the four important feature subsets and use the ensembled classifier to predict Phosphorylation.

### 3.1 Feature contribution

**FIGURE 2:**
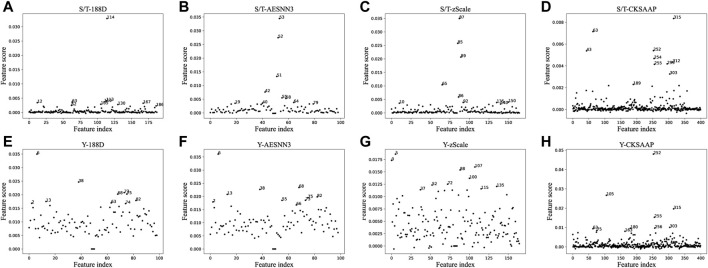
The scatter plots of feature importance using different feature extraction methods.

For each type of features, we divide the training data randomly for training the classifier and selecting the best classifier, and further calculate the importance score of the features. The results of feature importance scores for their S/T data are shown in Figure 2. The figure shows that only a small fraction of the proposed features have relatively high scores, such as feature $114_{th}$ of 188D, which has a 3% impact on the results. 188D, AESNN3 and zScale have a single feature that can have a maximum of 3% impact on the results, while CKSAAP can only had only 0.8% effect on the results. Some features have no effect on the results at all before and after modification, so we use feature selection to find effective features and reduce feature dimensionality at the same time.

Due to the large difference in the amount of data between S/T phosphorylation and Y phosphorylation, we take 30% of the sample classifiers for the S/T data and used 70% samples for testing to find the optimal classifier. And the Y data set is trained using 70% of the training set, 30% to find the optimal classifier.

### 3.2 Comparison of the different feature

**FIGURE 3:**
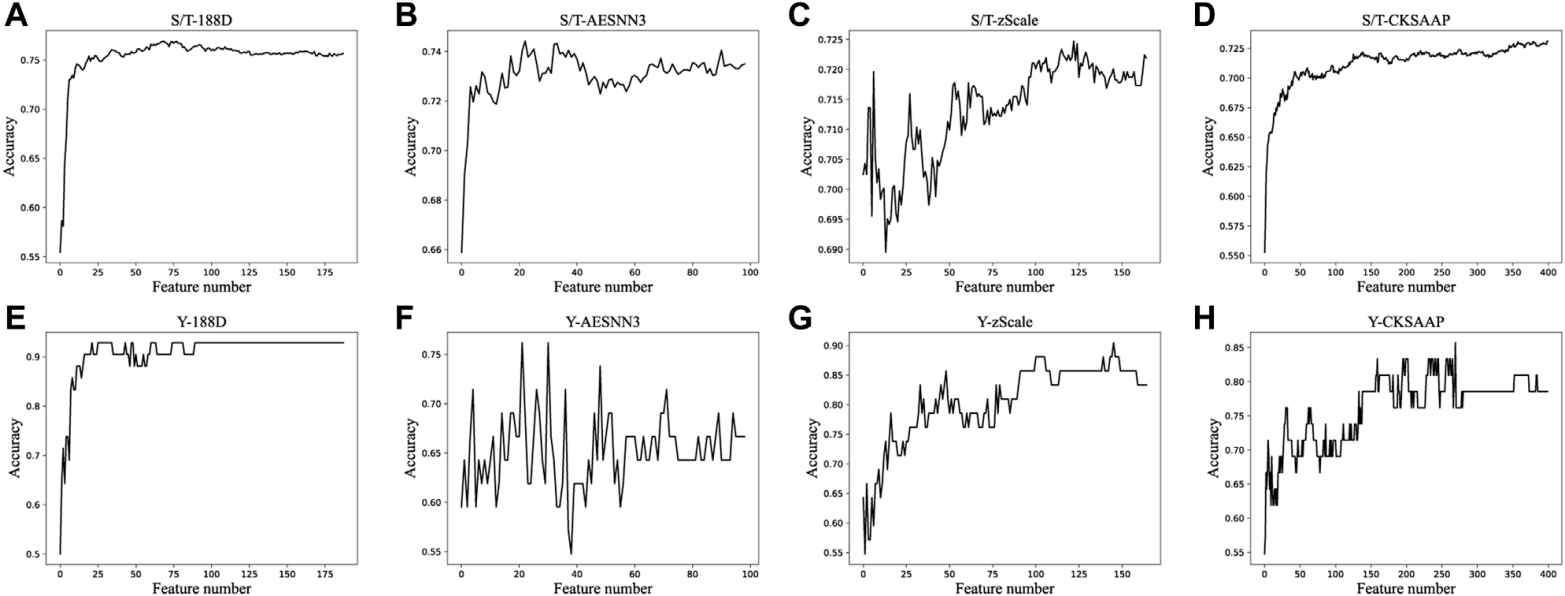
Accuracies derived from the incremental strategy using different feature extraction methods.

In order to remove the features that have no effect on the results, we stack the features in order of decreasing feature importance, and then randomly select 70% of the data set to train the ensembled classifier, after which the classification accuracy is calculated on the out-of-bag data set. The accuracy of the classifier is then plotted for each dimension of the S/T data, as shown in Figure 3. The figure shows that the accuracy of all features generally increases first during the process of superposition, and then remains stable around a certain value, and then the accuracy does not increase significantly as the features increase, and sometimes even decreases. And we choose the features whose accuracy rate just keeps stable. We select the top 35, 23, 139 and 122 most important features among 188D, AESNN3, CKSAAP and zScale features, respectively, and the number of features selected and their accuracy score are in Table 2 **.**

**TABLE 2:** Accuracy on the same independent test datasets of optimal feature subset.

Data type	Feature extract	Feature number	ACC(%)	SN (%)	SP (%)
S/T	188D	35	76.04	82.67	49.42
	AESNN3	23	74.42	72.01	76.83
	CKSAAP	139	72.20	78.18	66.27
	zScale	122	72.47	73.40	71.55
Y	188D	22	92.86	95.24	90.48
	AESNN3	22	76.19	71.43	80.95
	CKSAAP	196	83.35	80.95	85.71
	zScale	100	88.10	85.71	85.71

### 3.3 Comparison of the different method

After we select the optimal subset of each type of features based on the classification accuracy curve of Figure 3, we splice the selected subset of features, where 340 dimensional features are obtained for S/T data and 340 features are obtained for Y data. After that we use the random forest classifier for predicting the samples. We use all the training data to train the classifier and then come up with an independent test set for prediction. Then we compare with other methods on the same dataset, the results are shown in Table 3.

**TABLE 3:** Compared with other methods on the same independent test datasets.

Data type	Method	ACC (%)	SN (%)	SP (%)
S/T	DeepIPs	80.63	79.61	83.50
	DeepPSP	80.21	76.65	83.78
	MusiteDeep2017	80.17	78.87	81.46
	Our method	80.81	75.25	86.38
Y	DeepIPs	83.33	90.48	80.95
	DeepPSP	76.19	95.24	57.14
	MusiteDeep2017	80.95	85.71	76.19
	Our method	95.24	100	90.48

As can be seen from Table 3, the classification accuracy of our method is similar to that of the method (Wang et al., 2017; Guo et al., 2020; Lv et al., 2021) on the S/T dataset. On the Y data set it can be seen that the classification accuracy of our method is much higher than that of the deep learning-based method. This is due to the fact that there is a larger amount of S/T and the neural network can train the parameters better, but for Y data, there are only 204 data, which is not enough for the deep learning model to converge. This also shows the drawback of neural networks, which cannot train a good model when the amount of data is small.

## 4 Discussion

By analyzing the extracted features, we find that the classification accuracy is low when using the optimal subset of features extracted from a single type of features, such as the S/T data set are at 75%, while when we splice the optimal subset of four types of features, the classification accuracy can reach 80.81%. This also shows that when detecting phosphorylation site, the features are not well identified when using one type feature alone using machine learning for classification. When we ensemble multiple features, it is possible to capture the features that have an important role.

From Table 3, the deep learning methods are based only on data-driven for encoding protein sequences (have on functional knowledge of the protein), and although these methods achieve more than 80% accuracy on both S/T data sets, this is only because of the large amount of S/T data that allows the neural network to learn associations between features autonomously. However, for Y data, we find that the accuracy of the neural network is far behind that of our proposed method. And when we use only the first 22 of 188D features, we can achieve 92.86% accuracy. This also illustrates the effectiveness of our method of extracting features even using knowledge-driven and data-driven extraction of effective features.

While for the most important features found for example in the S/T dataset 188D features, the most important features are the last, first and the third quartile one of the features which is the positive, respectively. This indicates that the positive charge property of SARS-COV-2 positive samples occupies an important position in the classification of phosphorylation sites. The $21_{th}$ and $22_{th}$ features also illustrates the hydrophobicity of SARS-COV-2 protein. This is also consistent with the study by Gao et al. (2021). The remaining important features are the $63_{th}$ which is amino acid composition with Polar polarity at 8.0–9.2, and proline amino acid content, the $167_{th}$ which is the burned category of solvent accessibility, the $105_{th}$ which is the neutral category of charge, the $62_{th}$ which is amino acid composition with polar polarity at 4.9–6.2, *etc.*

In contrast, sequence-based features, such as CKSAAP and zScale features, are not suitable for short sequence amino acid feature processing because the short amino acid sequences result in a large number of features of this type with zero.

## 5 Conclusion

This study uses a computational biology approach to explore the nature of phosphorylation of SARS-COV-2 to make a small contribution to SARS-COV-2 drug discovery. In this study, an ensembled learning-based feature selection method is proposed that combines knowledge-driven and data-driven approaches to find out the important features for protein phosphorylation site prediction, then which a subset of important features based on heavier amino acid feature extraction rules are spliced for prediction. Comparing with other neural network-based methods, the results show that our method can not only obtain high accuracy on small samples, but also find biological features related to phosphorylation sites. This also indicates the accuracy, reliability and interpretability of our method. Most importantly the model is built to be of particular value in predicting the phosphorylation sites in host cells infected with SARS-COV-2.

## Data Availability

The original contributions presented in the study are included in the article/supplementary material, further inquiries can be directed to the corresponding author.
